# Inflammatory Biomarkers in Chronic Pain Predict Psychiatric Morbidity: A Multi-Network EHR Cohort

**DOI:** 10.1192/j.eurpsy.2026.11475

**Published:** 2026-07-31

**Authors:** A. S. Verma, V. Sharma, A. Pathak, R. Gupta

**Affiliations:** 1 Psychiatry, Case Western Reserve University/ Metrohealth, Cleveland, United States; 2 Psychiatry, Dayanand Medical College & Hospital, Ludhiana, India; 3 Psychiatry, Cleveland Clinic Main Campus, Cleveland; 4 Psychiatry, Brigham & Women’s Faulkner Hospital; 5 Psychiatry, Harvard Medical School, Boston, United States

## Abstract

**Introduction:**

There is an increased recognition of the role of systemic inflammation, mediated through inflammatory markers like IL-6, CRP, in pathophysiology of psychiatric disorders like depression, schizophrenia and substance abuse. While bidirectional influence of chronic pain and psychiatric morbidity is also understood, large-scale, real-world estimates on this topic remain limited. We aim to bridge this gap through a cohort study using TriNetX.

**Objectives:**

To compare 5-year risk of major depressive disorder (MDD), suicidality, and substance use disorder (SUD) in chronic pain patients with high vs. low inflammatory biomarkers.

**Methods:**

We used the TriNetX US Collaborative Network (71 health systems). Adults (≥18y) with chronic pain (ICD-10: G89.21, G89.28, G89.29, G89.4, M79.7) were stratified by inflammatory status using most-recent lab thresholds, each with ≥2 instances: high inflammation (ESR ≥20 mm/h [LOINC 4537-7], CRP ≥3 mg/L [1988-5], hs-CRP ≥3 mg/L [30522-7], or haptoglobin ≥200 mg/dL [4542-7]) vs. low (≤ these thresholds). Index was first date meeting pain + lab criteria; outcomes were assessed from day 1 to 5 years. Propensity-score matching (1:1) balanced demographics (642,785 per group post-match). We ran risk analyses excluding prior outcomes and Kaplan–Meier with log-rank tests and hazard ratios (Table 1)

**Results:**

After matching, MDD risk over 5 years was 19.2% in the high-inflammation cohort vs. 17.9% in low-inflammation (risk ratio [RR] = 1.074, 95% CI 1.064–1.083; HR = 1.162, 95% CI 1.151–1.174; log-rank p<0.001). Suicidality risk was 2.1% vs. 1.8% (RR = 1.170, 95% CI 1.141–1.199; HR = 1.247, 95% CI 1.216–1.279; p<0.001). SUD risk was 11.5% vs. 10.3% (RR = 1.112, 95% CI 1.100–1.125; HR = 1.188, 95% CI 1.174–1.203; p<0.001) (Table 2). Absolute risk differences were +1.3% (MDD), +0.3% (suicidality), and +1.2% (SUD).

**Image 1:**
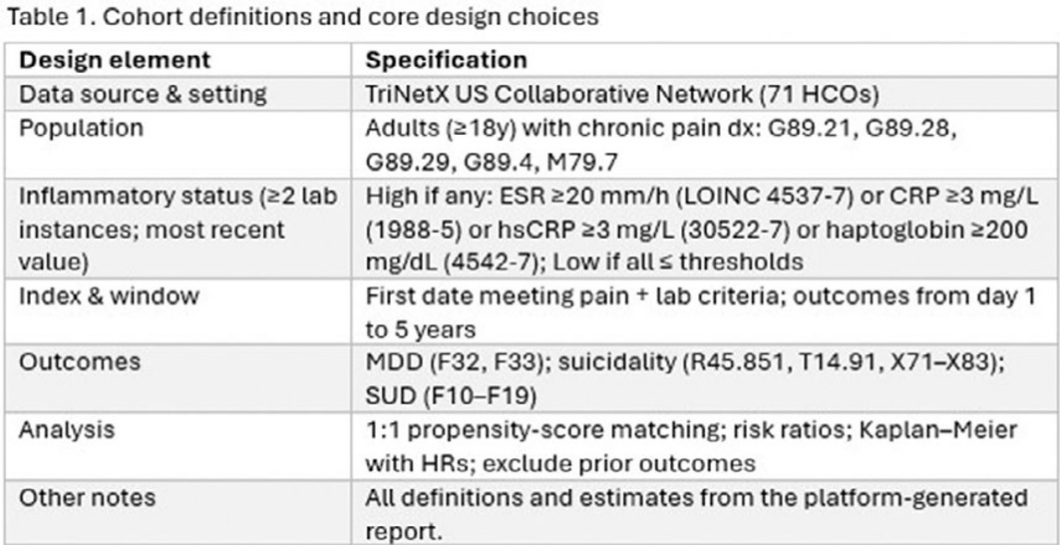
Image 1: Long description.

**Image 2:**
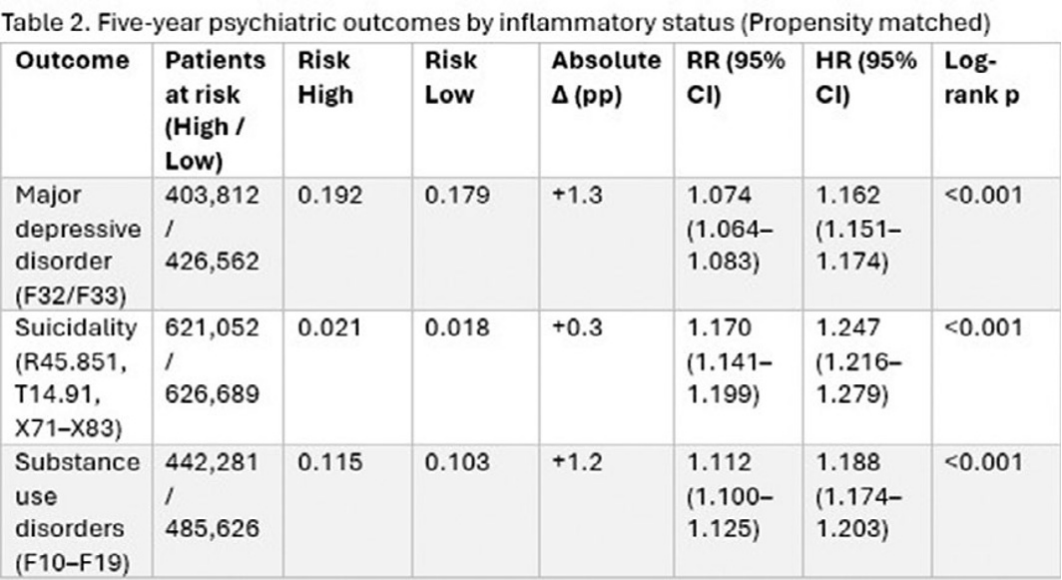
Image 2: Long description.

**Conclusions:**

Among adults with chronic pain, elevated inflammatory biomarkers are associated with higher 5-year incidence of MDD, suicidality, and SUD. Findings support routine mental-health screening when inflammatory markers are elevated and motivate trials testing anti-inflammatory or immunomodulatory strategies alongside standard psychiatric care. Observational design and EHR limitations (measurement variability, residual confounding) apply.

**Disclosure of Interest:**

None Declared

## Long descriptions

### Image 1: Long description

The table consists of seven rows across two columns: Design element and Specification.

* Data source and setting: TriNetX U S Collaborative Network (71 H C Os).

* Population: Adults (greater than or equal to 18y) with chronic pain dx: G 89.21, G 89.28, G 89.29, G 89.4, M 79.7.

* Inflammatory status (greater than or equal to 2 lab instances; most recent value): High if any: E S R greater than or equal to 20 mm/h (L O I N C 4537-7) or C R P greater than or equal to 3 mg/L (1988-5) or hs C R P greater than or equal to 3 mg/L (30522-7) or haptoglobin greater than or equal to 200 mg/dL (4542-7); Low if all less than or equal to thresholds.

* Index and window: First date meeting pain + lab criteria; outcomes from day 1 to 5 years.

* Outcomes: M D D (F 32, F 33); suicidality (R 45.851, T 14.91, X 71–X 83); S U D (F 10–F 19).

* Analysis: 1:1 propensity-score matching; risk ratios; Kaplan–Meier with H Rs; exclude prior outcomes.

* Other notes: All definitions and estimates from the platform-generated report.

Navigate back to Image 1:.

### Image 2: Long description

The table contains eight columns: Outcome, Patients at risk (High / Low), Risk High, Risk Low, Absolute Delta (p p), R R (95% C I), H R (95% C I), and Log-rank p.

* Major depressive disorder (F 32 / F 33): Patients at risk are 403,812 High / 426,562 Low. Risk High is 0.192 and Risk Low is 0.179. Absolute Delta is +1.3. R R is 1.074 (1.064–1.083). H R is 1.162 (1.151–1.174). Log-rank p is less than 0.001.

* Suicidality (R 45.851, T 14.91, X 71–X 83): Patients at risk are 621,052 High / 626,689 Low. Risk High is 0.021 and Risk Low is 0.018. Absolute Delta is +0.3. R R is 1.170 (1.141–1.199). H R is 1.247 (1.216–1.279). Log-rank p is less than 0.001.

* Substance use disorders (F 10–F 19): Patients at risk are 442,281 High / 485,626 Low. Risk High is 0.115 and Risk Low is 0.103. Absolute Delta is +1.2. R R is 1.112 (1.100–1.125). H R is 1.188 (1.174–1.203). Log-rank p is less than 0.001.

Navigate back to Image 2:.

